# Antiplatelet Activity of Natural Bioactive Extracts from Mango (*Mangifera Indica* L.) and its By-Products

**DOI:** 10.3390/antiox8110517

**Published:** 2019-10-29

**Authors:** María Elena Alañón, Iván Palomo, Lyanne Rodríguez, Eduardo Fuentes, David Arráez-Román, Antonio Segura-Carretero

**Affiliations:** 1 Area of Food Technology, Regional Institute for Applied Scientific Research (IRICA), University of Castilla-La Mancha. Avda. Camilo José Cela, 10, 13071 Ciudad Real, Spain; 2 Department of Analytical Chemistry, Faculty of Sciences, University of Granada, C/Fuentenueva s/n, 18071 Granada, Spain; darraez@ugr.es (D.A.-R.); ansegura@ugr.es (A.S.-C.); 3 Research and Development of Functional Food Centre (CIDAF), PTS Granada, Avda. Del Conocimiento 37, Bioregión Building, 18016 Granada, Spain; 4 Thrombosis Research Center, Department of Clinical Biochemistry and Immunohaematology, Faculty of Health Sciences, Interdisciplinary Center on Aging (CIE), University of Talca, 3460000 Talca, Chile; Lyannerodriguez89@gmail.com (L.R.); efuentes@utalca.cl (E.F.)

**Keywords:** Mango, by-products, antiplatelet activity, bioactive compounds, HPLC-DAD-q-TOF-MS

## Abstract

The potential antiplatelet aggregation effects of mango pulp and its by-products (peel, husk seed, and seed) due to the presence of bioactive compounds were explored. Among them, mango seed exhibited a 72% percentage inhibition of platelet aggregation induced by adenosine 5’-diphosphate (ADP) agonist with a demonstrated dose-dependent effect. This biological feature could be caused by the chemical differences in phenolic composition. Mango seed was especially rich in monogalloyl compounds, tetra- and penta-galloylglucose, ellagic acid, mangiferin, and benzophenones such as maclurin derivatives and iriflophenone glucoside. Mangiferin showed an inhibitory effect of 31%, suggesting its key role as one of the main contributors to the antiplatelet activity of mango seed. Therefore, mango seed could be postulated as a natural source of bioactive compounds with antiplatelet properties to design functional foods or complementary therapeutic treatments.

## 1. Introduction

Cardiovascular diseases (CVD) have the highest mortality rate of all types of diseases worldwide. Atherosclerosis and thrombotic processes associated with the rupture of vulnerable plaques are the main triggers of cardiovascular and cerebrovascular strokes [1,2]. It has been determined that platelets represent the bridge between inflammation and thrombosis, which are fundamental processes in the development of atherothrombosis [3]. The general pathogenies entails platelet activation, subsequent adhesion, release of granule content, and platelet aggregation [4]. The multiple mechanisms of action and the side effects of drugs make the research on natural bioactive compounds useful for pharmaceutical constituents in the prevention or complementary treatment of antiplatelet therapy.

The antiplatelet activity of numerous bioactive compounds detected in fruits and vegetables and their multiple mechanisms of actions have recently been highlighted [5]. However, these bioactive compounds can not only be found in the edible fraction but also in their by-products. This fact has made by-products a profitable niche of phytochemicals to be used as functional substances [6], although some of their characteristics such as bioavailability, pharmakinetic, pharmacodynamics properties, safety, and toxicity still remain unclear. 

In this context, mango fruit (*Mangifera indica* L) is the crop with the second highest production and acreage requirements, behind only bananas [7]. Although it is mainly consumed fresh, plenty of mango-derived products have been created such as juice, nectar, purée, ice cream, jam, canned slices, chutneys, etc. Consequently, by-products such as peel, seed, and seed husk, constituting 35%–60% of the fruit [8], are generated during the industrial processes. Recently, the high content of health-enhancing compounds like polyphenols, anthocyanins, and carotenoids in mango by-products has been reported [9]. Thus, different scientific investigations have been focused on the revalorization of mango by-products due to their functional properties and potential therapeutic uses of their bioactive compounds [10,11].

Recently, the antioxidant and anti-inflammatory effects of phenolic compounds obtained from mango by-products and water extracts by reducing the nitric oxide (NO) levels produced by lipopolysaccharides (LPS)-stimulated macrophages have been reported [12]. This biological activity postulates mango by-products to enhance cardiovascular health, and makes other physiological effects of mango by-products plausible against the pathogenies of cardiovascular diseases. 

Therefore, the main goal of this work is to explore the inhibitory effects of platelet aggregation of extracts from mango pulp and its by-products (peel, husk seed, and seed) and characterize the phenolic composition in order to figure out the compounds responsible for the antiplatelet activity. 

## 2. Materials and Methods 

### 2.1. Fruit Material and Sample Preparation

About 20 kg of Keitt mangos cultivated in the Tropical Coast of Granada were provided by Miguel García Sanchez e Hijos S.A. (Motril, Spain) at the optimum maturation stage (13.1–16.0 °Brix) in November 2016. Once they had arrived at the laboratory, samples were cleaned and the different parts of mango (pulp, peel, seed, and seed husk) were separated manually and cut into small and homogenous pieces. To preserve chemical composition of mango pulp and its by-products, samples were submitted to a lyophilization process (Advantage Plus EL-85 freeze dryer, SP Scientific, Ipswich, Suffolk, UK). Then each sample was milled (IKA M20-IKAWERKE GmbH & Co. KG, Staufen, Germany), homogenized, and stored at −18 °C prior to their analyses.

### 2.2. Isolation of Extracts Rich in Polyphenols from Mango and its By-Produts

#### 2.2.1. Extracts for Anti-Platelet Aggregation Activity Assay

The isolation of polyphenols from samples was carried out following an adapted method from Gómez-Caravaca et al., 2016 [13]. For each sample, 2 g of freeze-dried powder were sonicated for 15 min with 40 mL of a solution of methanol/water (80:20 v/v). After the extraction process, the mixture was centrifuged for 15 min at 7700 g and at 4 °C. The supernatant was removed and other two consecutive extraction steps were repeated. All supernatants were collected, evaporated to dryness, and stored at −18 °C. With the aim of obtaining arond 2 g of each extract, we performed the antiplatelet activity and took into account the yield of each extraction (pulp, 79.4%; peel, 44.6%; seed husk, 7.4%, and husk, 16.7%). In total, 4 g of pulp, 6 g of peel, 32 g of seed husk, and 16 g of husk were processed using the previous extraction procedure. 

#### 2.2.2. Extracts for Analytical Characterization 

For each samples (pulp, peel, seed husk, and seed) 0.5 g of freeze-dried powder were extracted with 10 mL of methanol/water (80:20 v/v) under sonication for 15 min [13]. After the extraction process, mixture was centrifuged for 15 min at 7700 g and at 4 °C. The supernatant was removed and the extraction step was then repeated twice. Finally, all supernatants were also collected, evaporated, reconstituted in 3 mL of methanol/water (80:20 v/v), filtered (0.2 µM, Milllipore), and stored at −18 °C. All extractions were performed in duplicate.

### 2.3. Human Platelet Isolation

Platelet-rich plasma (PRP) was obtained from six healthy young volunteers who had previously signed a consent report. Samples were extracted by phlebotomy in 3.2% sodium citrate tubes (Becton Dickinson Vacutainer Systems, Franklin Lakes, NJ, USA). Then, blood samples were centrifuged (DCS-16 Centrifugal Presvac RV) at 240 g for 10 min to obtain platelet-rich plasma (PRP). Two-thirds of PRP was removed and centrifuged at 650 g for 10 min. The platelet count was performed in a blood count (Bayer Advia 60 Hematology System, Tarrytown, NY, USA).

### 2.4. Anti-Platelet Aggregation Activity Assay

The anti-platelet aggregation activity of mango and its by-products was evaluated by a turbidimetric method [14] using a lumi-aggregometer (Chrono-Log, Haverton, PA, USA). The PRP (200 × 10^9^ platelets/L) was preincubated with 20 μL of phosphate-buffered saline, PBS, (negative control: maximum aggregation), or power extracts from each part of the mango (1 mg/mL) for 3 min at 37 °C. In order to evaluate the dose-dependent effects against platelet aggregation, different concentrations (0.1, 0.5, and 1 mg/mL) of the more active extract were tested as well as the antiplatelet properties of some standards compounds such as mangiferin (Sigma-Aldrich, St. Louis, Missouri, MO, USA) at 1 mg/mL. For all assays, platelet aggregation was induced by adding adenosine 5’-diphosphate (ADP, 4 μM) supplied by Sigma-Aldrich (St. Louis, Missouri, MO, USA) as an agonist. Platelet aggregation was measured by triplicate as the increase in light transmission occurred for 6 min and results were expressed as a percentage of inhibition of aggregation. 

### 2.5. Phenolic Characterization of Extracts by HPLC-DAD-q-TOF-MS

Power extracts from the extraction of 0.5 g of samples were redissolved in 3 mL of methanol/water (80:20 v/v). After their filtration, extracts were analyzed by HPLC-DAD-q-TOF-MS (Agilent 1200 series coupled to 6540 Agilent Ultra-High-Definition Accurate-Mass q-TOF-MS, Agilent Technologies, Palo Alto, CA, USA) according to the method proposed by Gómez-Caravaca et al. [13]. 

Identification was performed based on relative retention times, UV-Vis spectra, and mass spectra obtained by q-TOF-MS and from the literature. Quantification was carried out using calibration standards curves of gallic acid, coumaric acid, ferulic acid, vanillic acid, catechin, quercetin, ellagic acid, and mangiferin (Sigma Aldrich, St. Louis, MO, USA). Spectral and chromatographic data are compliled in Supplementary Table S1. Integration and data processing were performed using Mass Hunter Workstation Software, Qualitative Analysis, version B.07.00 (Agilent Technologies, Inc. 2014).

### 2.6. Statistical Analysis

The IBM SPSS statistics v.22.0 for Windows statistical package was employed to carry out statistical analysis. Anti-platelet aggregation data set was submitted to the ANOVA analysis and the Tukey’s post-hoc test, while the Student-Newman-Keuls test was applied to the chemical data in order to determine the significant differences between samples. 

## 3. Results and Discussion

### 3.1. Anti-Platelet Aggregation Activity of Mango and its By-Products

**Figure 1:**
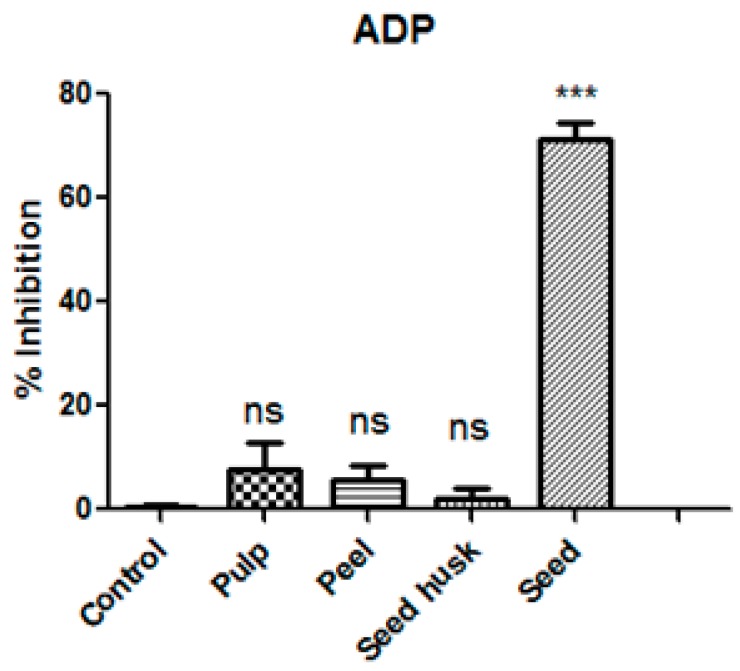
Antiplatelet aggregation activity results of extracts from mango and its by-products (peel, seed husk and seed) at 1.0 mg/mL against adenosine 5’-diphosphate (ADP) agonist (4 μM) expressed as mean value of inhibition percentages (*n* = 6). Data was analyzed using ANOVA of one factor. Post hoc analyses were conducted using Tukey’s test. *** denotes significant differences compared to the negative control (absence of extract) at *p* = 0.001; ns denotes no statistical differences.

The anti-platelet aggregation activity of extracts from edible part of mango (pulp) and its by-products (peel, seed husk, and seed) was evaluated on a human platelet whose aggregation was induced by using ADP (4 μM) as an agonist. Inhibition percentages of platelet aggregation of extracts from mango and its by-products under the optimum maturation stage at a concentration of 1.0 mg/mL are shown in Figure 1. It can be observed that human platelet aggregation triggered by the ADP agonist was not significantly inhibited by pulp, skin, and husk seed extracts compared to the negative control. However, mango seed extract exhibited a significantly higher inhibition percentage, which was around 72%. These findings can hardly be compared with others due to the scarce bibliography regarding the antiplatelet activity of mango by-products. Recently, the influence of the ripening stage of mango peel on rat platelet aggregation has been reported [15]. Ripe fruit peel extract showed better platelet aggregation inhibitory effects. The inhibition percentage observed in ripened mango peel water extract at 0.8 mg/mL in rat platelets was considerably higher (approximately 25%) in comparison with our results tested against human platelet (approximately 10%). To the best of our knowledge, anti-platelet aggregation data on mango pulp and their by-products on human blood have not been reported until now.

**Figure 2:**
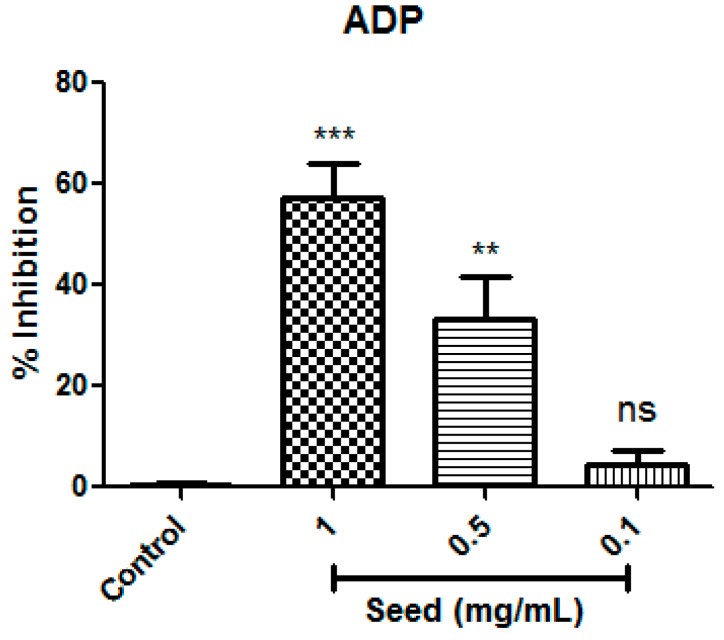
Study of the dose-dependent effect of mango seed extract on antiplatelet aggregation activity against ADP agonist (4 μM) expressed as a mean value of inhibition percentages (*n* = 6). Data were analyzed using ANOVA of one factor. Post hoc analyses were conducted by Tukey’s test, ** and *** denotes significant differences at *p* = 0.01 and *p* = 0.001, respectively; ns denotes no statistical differences.

Therefore, based our results, mango seed extract was shown to be a potential inhibitor of human platelet aggregation induced by ADP agonist at 1.0 mg/mL with an inhibition percentage of 72%. The current study also examined the dose-dependent effects of mango seed extract on inhibition of platelet aggregation. To investigate the dose-dependent activity, several concentrations of mango seed extract (1.0, 0.5 and 0.1 mg/mL) were tested against the platelet aggregation induced by ADP agonist (4 μM). Significant differences in antiplatelet aggregation activity were identified between the highest mango seed extract (1.0 mg/mL) and the middle dose (0.5 mg/mL) at *p* = 0.001 and *p* = 0.01, respectively (Figure 2). Meanwhile no significant differences were found among the negative control and the lowest dose tested (0.1 mg/mL). Therefore, data showed a clear dose-dependent effect that increased the inhibition of platelet aggregation when the mango seed extract concentration increased.

### 3.2. Phenolic Characterization of Extracts by HPLC-DAD-q-TOF-MS

The nutraceutical and pharmaceutical significance of mango to human health has been attributable to its phenolic composition [11]. Therefore, a phenolic characterization of the edible and non-edible parts of mango was carried out with the aim to relate chemical differences with the antiplatelet activity observed by mango seed. A large number of phenolic compounds with different natures and structures were detected in mango pulp and its by-products. The most abundant and numerous family found in mango was gallic acid derivatives (Table 1). The characteristic high content of gallic acid of mango fruit and its by-products in comparison with other tropical fruits has already been determined previously [12,16].

Mango seed was particularly rich in monogalloyl compounds compared to the rest of mango parts, especially edible mango fraction and seed husk by-product. The sum of the total monogalloyl compounds reached the value of 720.30 ± 2.27 mg/100 g_dry matter_ in mango seed. In contrast, considerable lower concentrations of total monogalloyl compounds were found in pulp, peel, and seed husk (52.18 ± 0.10, 235.34 ± 11.07, and 68.15 ± 3.48 mg/100 g_dry matter_, respectively). It was noteworthy that the highest content of methylgallate was observed in mango seed (558.86 ± 6.74 mg/100 g_dry matter_), which was the major phenolic compound detected in this matrix. The strong antioxidant power of this compound has already been reported in the bibliography [10]. Significantly higher concentrations of other monogalloyl compounds such as gallic acid, galloyl diglucoside, and galloylquinic acid in mango seed were also found. Higher quantities of galloylglucose were found in mango peel, as were monogalloyl derivatives. On the other hand, both edible mango fraction and seed husk exhibited the lowest values of monogalloyl compounds compared to peel and seed by-products. These findings were in good agreement with those reported by other authors, who also detected higher concentrations of gallic acid and methylgallate in mango seed and found more abundant galloylglucose in mango peel [13].

Structurally, gallic acid contains hydroxyl groups and a carboxylic acid group, so its molecules have the ability to react with one another to generate digalloyl compounds. Regarding these compounds, a contrary behavior was evidenced since the highest concentrations of digalloylglucose, methyldigallate, and digalloylquinic acid were found in mango peel (Table 1). Consequently, peel was found to be the fraction of mango with the greatest digalloyl content (137.98 ± 4.09 mg/100 g_dry matter_) in comparison with the rest of samples whose composition on digalloyl compounds ranged between 8.13 and 22.50 mg/100 g_dry matter_).

The occurrence of gallotannins, hydrolyzable tannins, was also detected in mango pulp and its by-products. Indeed, mango is recognized as one of the fruit with the major content of gallotannins [17]. Gallotannins contain gallic acid substituents esterified with glucose whose galloylation reaction yields tri-, tetra-, penta-, hexa-, and hepta-galloylglucoses (Table 1). Gallotannins concentrations varied according to the fraction of mango considered. Pulp and seed husk exhibited the poorest content of gallotannis (13.37 ± 0.02 and 25.47 ± 0.52 mg/100 g_dry matter_, respectively) meanwhile peel and seed presented considerably higher total amounts (182.90 ± 3.62 and 332.66 ± 13.32 mg/100 g_dry matter_, respectively). Mango peel was characterized by the significant concentrations of tri- and hepta-galloylglucose compared to the rest of the samples. On the other hand, mango seed resulted to be especially rich in tetra- and penta-galloylglucose. The high concentration of penta-galloylglucose in mango seed (177.31 ± 2.41mg/100 g_dry matter_), which also was observed by other authors [13], should be noted due to its multiple functional properties such as antioxidant, anti-cancer, anti-viral, anti-microbial, anti-inflammatory, and anti-diabetic activities [18]. However, from a functional point of view, it should be taken into account that seed content in penta-galloylglucose could decrease drastically during the maturation process, as has been recently reported for mango pulp [19].

A large number of phenolic acid compounds derived from vanillic acid, hydroxybenzoic acid, coumaric acid, ferulic acid, and sinapic acid were also found in mango pulp and its by-products (Table 2). The most abundantly occurring phenolic acid derivative was vanillic acid glucoside whose concentrations in mango peel and seed were significant higher than in mango pulp and seed husk. Based on the results, mango peel exhibited the major amounts of phenolic acid derivative compounds. However, only the presence of vanillic acid glucoside, *p*-hydroxybenzoic acid glucoside, and ferulic acid hexoside on mango seed was confirmed. Therefore, phenolic acid derivative compounds seemed to not play an important role in the antiplatelet activity of mango seed extract.

Other compounds of different nature and structure were also detected in mango samples (Table 3). Regarding ellagic acid and catechin, pulp and seed husk presented the lowest values, while seed was the mango fraction with the highest amount of ellagic acid (7.46 ± 0.54 mg/100 g_dry matter_). The quantity of ellagic acid in mango seed extract had been reported from 3 to 156 mg equivalents of gallic acid per 100 g depending on the extraction method [20]. Several studies have proven the strong radical-scavenging activity of ellagic acid, even at very low concentrations [21,22]. Quercetin was also detected in glycosides, with the most common forms found being quercetin glucoside, quercetin galactoside, quercetin xyloside, and quercetin arabinopyranoside (Table 3). These flavonols were almost exclusively found in mango peel, which was in good agreement with findings reported by other authors [13,23]. Only small quantities of quercetin glucoside were detected in seed husk and seed. 

**Figure 3:**
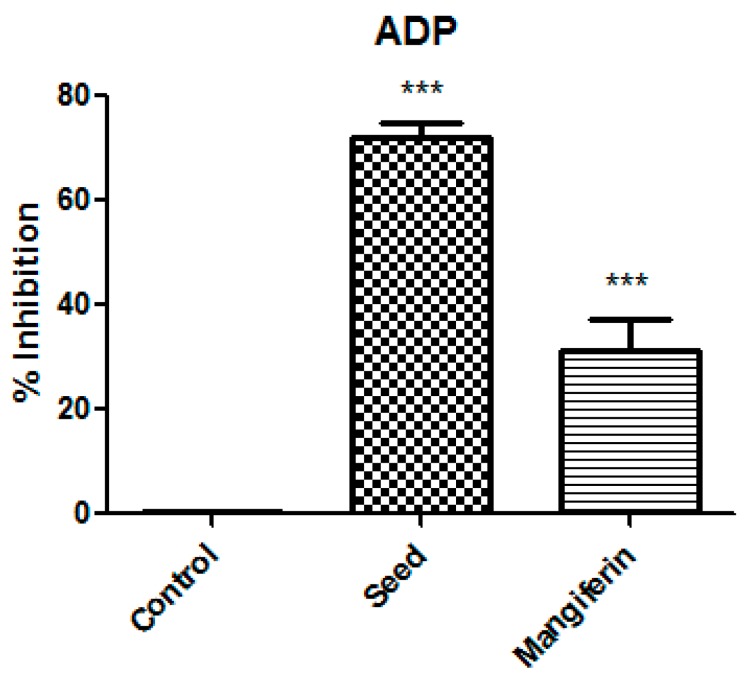
Antiplatelet aggregation activity results of mango seed extract and mangiferin at 1.0 mg/mL against ADP agonist (4 μM) expressed as mean value of inhibition percentages (*n* = 6). Data was analyzed using ANOVA of one factor. Post hoc analyses were conducted by Tukey’s test. *** denotes significant differences compared to the negative control (absence of extract) at *p* = 0.001; ns denotes no statistical differences.

On the other hand, the phenolic fraction of mango seed was characterized by the highest concentrations of 7-*O*-galloyltricetilflavan, mangiferin, and benzophenones such as maclurin glucoside, maclurin galloyl glucoside, maclurin digalloyl glucoside, and iriflophenone glucoside (Table 3). Among them, the most striking differences were those found on mangiferin content (1,3,6,7-tetrahydroxyxanthone-C2-β-D-glucoside). Mango seed showed the highest quantities of mangiferin (148 ± 0.74 mg/100 g_dry matter_). Only small amounts of mangiferin were found in seed husk, while its occurrence was not detectable in pulp and peel. These results were in good agreement with those reported in bibliography, which also regarded mango seed as the part of mango with the highest mangiferin content [13,24]. Furthermore, the content of mangiferin in mango seed appeared to be influenced by the cultivar, as cv.*Keitt* presented major quantities of mangiferin in comparison with other varieties such as *Osteen* or *Sensación* [24]. The abundance of mangiferin in mango seed should be noted since mangiferin has been highlighted as a multi-target bioactive compound due to its health-endorsing properties. Indeed, several therapeutic and cosmetic applications have been recently attributed to mangiferin due to its antioxidative, antiaging, antidiabetic, anti-tumor, neuropropetective, cardiovascular, immunomodulatory and hepatoprotective effects, among others [25,26]. Consequently, the anti-platelet aggregation activity of mangiferin (1.0 mg mL^−1^) on human platelet whose aggregation was induced by ADP (4 μM) was also estimated. Results showed that mangiferin has an important anti-platelet effect, exhibiting 31% inhibition (Figure 3). However, it has been demonstrated that for the same concentration, the antiplatelet aggregation activity increased more than two fold in mango seed. Therefore, although mangiferin showed a considerably bioactive effect, the antiplatelet activity of mango seed could also be explained by the action of other phenolic compounds and their possible synergistic interactions, or even by the presence of other compounds with reported bioactivity such as carbohydrates [27]. The percentage of carbohydrates reported in mango seed (64.24%) was considerably higher than that found in mango peel (31.24%) [28], which could have had an influence on the antiplatelet activity observed by mango seed. On the other hand, results compiled in bibliography also pointed out seed as the mango by-product with the highest antioxidant activity (28.92–32.61 g of Trolox equivalents per 100 g) compared to mango peel (5.39–6.01 g of Trolox equivalent per 100 g) evaluated by different in vitro methods [29]. Therefore, based our results and those found in other authors, mango seed seems to be an excellent source of phytochemicals with functional properties.

## 4. Conclusions

Mango seed was revealed as the fraction of the fruit with the most significant dose-dependent anti-platelet aggregation activity (inhibition percentage: 72%), compared to other mango by-products and the edible fraction. The chemical differences in phenolic composition found among different fractions of mango seemed to explain its bioactivity. Although mangiferin appeared to play a key role in this bioactivity, the antiplatelet effect of mango seed extract was not entirely explained by its action. Other compounds such as gallic acid, methylgallate and galloylquinic acid, tetra- and penta-galloylglucose, ellagic acid, 7-o-galloyltricetilflavan, iriflophenone glucoside, and maclurin C-glucoside and its derivatives were pointed out as contributors to antiplatelet aggregation activity. However, a comprehensive study of their individual antiplatelet properties should be addressed in further research. 

Taken together, these results highlighted the use of mango seed as a promising natural co-product with antiplatelet properties, which can be used as a pharmaceutical drug or as a functional food ingredient with therapeutic applications against platelet aggregation.

## Figures and Tables

**Table 1:** Mean concentration of gallic acid derivative compounds from different parts of *Keitt* mango variety at optimum maturation stage expressed as mg/100 g_dry matter_.

COMPOUNDS	PULP			PEEL			SEED HUSK			SEED		
	MEAN		SD	MEAN		SD	MEAN		SD	MEAN		SD
**Monogalloyl compounds**												
Gallic acid	1.55	±	0.02 ^a^	3.57	±	0.47 ^b^	3.93	±	0.24 ^b^	16.67	±	0.03 ^c^
Galloylglucose	42.36	±	0.23 ^a^	112.17	±	1.10 ^c^	43.09	±	1.83 ^a^	53.67	±	4.73 ^b^
Galloyl diglucoside	0.09	±	0.00 ^a^	5.69	±	0.07 ^b^	0.25	±	0.01^a^	6.64	±	0.11 ^c^
Methylgallate	5.41	±	0.48 ^a^	54.14	±	7.77 ^b^	14.43	±	0.89 ^a^	558.86	±	6.74 ^c^
Galloylquinic acid	2.76	±	0.12 ^a^	59.77	±	1.79 ^c^	6.45	±	0.50 ^b^	84.46	±	0.34 ^d^
**Digalloyl compounds**												
Digallic acid	ND			2.54	±	0.34 ^a^	1.67	±	0.00 ^a^	3.64	±	0.57 ^b^
Digalloylglucose	4.59	±	0.03 ^a^	31.17	±	0.57 ^c^	9.18	±	0.59 ^b^	4.23	±	0.09 ^a^
Methyl digallate	3.33	±	0.44 ^a^	27.80	±	4.88 ^c^	10.47	±	0.50 ^b^	ND		
Digalloylquinic acid	0.22	±	0.00 ^a^	76.48	±	0.55 ^d^	1.18	±	0.10 ^b^	2.22	±	0.02 ^c^
**Gallotannins**												
Trigalloylglucose	0.02	±	0.02 ^a^	9.07	±	0.04 ^d^	0.85	±	0.03 ^b^	3.26	±	0.05 ^c^
Tetragalloylglucose	1.64	±	0.02 ^a^	14.50	±	0.89 ^b^	3.16	±	0.13 ^a^	88.37	±	3.22 ^c^
Penta-galloylglucose	4.13	±	0.07 ^a^	26.61	±	0.91 ^b^	3.93	±	0.04 ^a^	177.31	±	2.14 ^c^
Hexagalloylglucose	4.00	±	0.08 ^a^	74.46	±	0.03 ^b^	9.78	±	0.25 ^a^	63.72	±	7.92 ^b^
Hepta-galloylglucose	3.59	±	0.00 ^a^	58.26	±	1.75 ^c^	7.75	±	0.15 ^b^	ND		

Values with different superscripts ^a,b,c,d^ in the same row denoted significant differences according to the Student-Newman-Keulstest at *p* ˂ 0.05. ND: not detected.

**Table 2:** Mean concentration of gallic acid derivative compounds from different parts of *Keitt* mango variety at optimum maturation stage expressed as mg/100 g_dry matter_.

COMPOUNDS	PULP			PEEL			SEED HUSK			SEED		
	MEAN		SD	MEAN		SD	MEAN		SD	MEAN		SD
Vanillic acid glucoside	3.90	±	0.19 ^a^	16.68	±	0.42 ^c^	7.94	±	0.36 ^b^	15.91	±	0.77 ^c^
*p*-Hydroxybenzoic acid glucoside	7.65	±	0.52 ^c^	7.27	±	0.20 ^c^	2.58	±	0.07 ^a^	6.06	±	0.18 ^b^
Dihydroxybenzoic acid glucoside	0.45	±	0.00 ^a^	0.36	±	0.01 ^a^	0.75	±	0.07 ^b^	ND		
Hydroxybenzoyl galloyl glucoside	0.26	±	0.00 ^b^	0.26	±	0.01 ^b^	0.07	±	0.01 ^a^	ND		
Coumaric acid glucoside	0.07	±	0.00 ^a^	0.52	±	0.01 ^c^	0.29	±	0.02 ^b^	ND		
Coumaroyl galloyl glucoside	0.29	±	0.02 ^a,b^	19.69	±	0.27 ^c^	0.61	±	0.00 ^b^	ND		
Ferulic acid hexoside	2.50	±	0.09 ^b^	1.81	±	0.01 ^a^	1.91	±	0.01 ^a^	2.91	±	0.22 ^c^
Sinapic acid hexoside	0.32	±	0.00 ^a^	0.97	±	0.00 ^b^	ND			ND		
Sinapic acid hexoside-pentoside	7.73	±	0.08 ^b^	14.16	±	0.07 ^c^	6.96	±	0.05 ^a^	ND		
Dihydro sinapic acid hexoside-pentoside	3.57	±	0.07 ^a^	4.90	±	0.08 ^b^	3.53	±	0.09 ^a^	ND		

Values with different superscripts ^a,b,c^ in the same row denoted significant differences according to the Student-Newman-Keuls test at *p* ˂ 0.05. ND: not detected.

**Table 3:** Mean concentration of ellagic acid, flavonols, xanthones, and benzophenones from different parts of *Keitt* mango variety at optimum maturation stage expressed as mg/100 g_dry matter._

COMPOUNDS	PULP			PEEL			SEED HUSK			SEED		
	MEAN		SD	MEAN		SD	MEAN		SD	MEAN		SD
Ellagic acid	0.02	±	0.00 ^a^	1.35	±	0.03 ^b^	0.56	±	0.04 ^a^	7.46	±	0.54 ^c^
Catechin	0.49	±	0.01 ^a^	12.18	±	0.78 ^c^	1.17	±	0.26 ^a^	9.97	±	0.71 ^b^
Quercetin glucoside	ND			21.28	±	0.63 ^c^	0.21	±	0.05 ^a^	1.39	±	0.14 ^b^
Quercetin galactoside	ND			10.91	±	0.45 ^b^	0.02	±	0.00 ^a^		ND	
Quercetin xyloside	ND			2.83	±	0.25 ^a^		ND			ND	
Quercetin arabinopyranoside	ND			2.94	±	0.20 ^a^		ND			ND	
Rhamnetin hexoside	ND			2.37	±	0.05 ^a^		ND			ND	
7-*O*-galloyltricetilflavan	ND			2.91	±	0.11 ^a^		ND		13.99	±	0.29 ^b^
Mangiferin	ND			ND			3.75	±	0.29 ^a^	148.12	±	0.74 ^b^
Maclurin C-glucoside	ND			ND			0.01	±	0.01 ^a^	11.40	±	0.48 ^b^
Maclurin galloyl glucoside	ND			4.60	±	0.04 ^b^	0.84	±	0.12 ^a^	9.63	±	0.04 ^c^
Maclurin digalloyl glucoside	ND			1.63	±	0.04 ^a^		ND		6.21	±	0.28 ^b^
Iriflophenone glucoside	ND			ND				ND		10.89	±	0.28 ^a^

Values with different superscripts ^a,b,c^ in the same row denoted significant differences according to the Studente-Newman-Keuls test at *p* ˂ 0.05. ND: not detected.

## Supplementary Materials

The following are available online at https://www.mdpi.com/2076-3921/8/11/517/s1, Table S1: Spectral and chromatographic data regarding to the bioactive compounds identified in mango and its by-products by HPLC-DAD-q-TOF-MS.

## References

[1] Badimon L., Vilahur G. (2008). Coronary Atherothrombotic Disease: Progress in Antiplatelet Therapy. Revista Esp. de Cardiol..

[2] Davies M.J. (2000). The pathophysiology of acute coronary syndromes. Heart.

[3] Geisler T., Anders N., Paterok M., Langer H., Stellos K., Lindemann S., Herdeg C., May A.E., Gawaz M. (2007). Platelet response to clopidogrel is attenuated in diabetic patients undergoing coronary stent implantation. Diabetes Care.

[4] Kong Y., Xu C., He Z.L., Zhou Q.M., Wang J.B., Li Z.Y., Ming X. (2014). A novel peptide inhibitor of platelet aggregation from stiff silkworm, Bombyx batryticatus. Peptides.

[5] Fuentes E., Palomo I. (2014). Antiplatelet effects of natural bioactive compounds by multiple targets: Food and drug interactions. J. Funct. Foods.

[6] Sagar N.A., Pareek S., Sharma S., Yahia E.M., Lobo M.G. (2018). Fruit and vegetable waste: Bioactive compounds, their extraction and possible utilization. Compr. Rev. Food Sci. F..

[7] Muchiri D.R., Mahungu S.M., Gituanja S.N. (2012). Studies on Mango (*Mangifera indica* L.) kernel fat of some Kenyan varieties in Meru. J. Am. Oil Chem. Soc..

[8] Ayala-Zavala J.F., Vega-Vega V., Rosas-Domínguez C., Palafox-Carlos H., Villa-Rodríguez J.A., Wasim Siddiqui M., Dávila-Aviña J.E., González-Aguilar G.A. (2011). Agro-industrial potential of exotic by-products as a source of food additives. Food Res. Int..

[9] Jahurul M.H.A., Zaidul I.S.M., Ghafoor K., Al-Juhaimi F.Y., Nyam K.-L., Norulaini N.A.N., Sahena F., Mohd Omar A.K. (2015). Mango (*Mangifera indica* L.) by-products and their valuable components: A review. Food Chem..

[10] Asif A., Farooq U., Akram K., Hayat Z., Shafi A., Sarfraz F., Sidhu M.A.I., Rehman H., Aftab S. (2016). Therapeutic potentials of bioactive compounds from mango fruit waste. Trends Food Sci. Tech..

[11] Massibo M., He Q. (2008). Major mango polyphenols and their potential significance to human health. Compr. Rev. Food Sci. F..

[12] Albuquerque Cavalacanti de Albuquerque M., Levit R., Beres C., Bedani R., De Moreno de LeBlanc A., Saad S.M.I., LeBlanc J.G. (2019). Tropical fruit by-products water extracts as sources of soluble fibers and phenolic compounds with potential antioxidant, anti-inflammatory and functional properties. J. Funct. Foods.

[13] Gómez-Caravaca A.M., López-Cobo A., Verardo V., Segura-Carretero A., Fernández-Gutiérrez A. (2016). HPLC-DAD-q-TOF-MS as a powerful platform for the determination of phenolic and other polar compounds in the edible part of mango and its by-products (peel, seed and seed husk). Electrophoresis.

[14] Fuentes E.J., Astudillo L.A., Gutierrez M.I., Contreras S.O., Bustamante L.O., Rubio P.I., Moore-Carrasco R., Alarcon M., Fuentes A., Gonzalez J.A. (2012). Fractions of aqueous and methanolic extracts from tomato (*Solanum lycopersicum* L.) present platelet antiaggregant activity. Blood Coagul. Fibrinolysis.

[15] Sai Srinivas S.H., Maneesh Kumar M., Prasada Rao U.J.S. (2017). Studies on isolation and antioxidant properties of bioactive phytochemicals from mango peel harvested at different developmental stages. Int. J. Agric. Environ. Bio-res..

[16] Siriamornpun S., Kaewseejan N. (2017). Quality, bioactive compounds and antioxidant capacity of selected climateric fruits with relation to their maturity. Sci. Hortic..

[17] Smeriglio A., Barreca D., Bellocco E., Trombetta D. (2017). Proanthocyanidins andhydrolyzable tannins: Occurrence, dietary intake and pharmacological effects. Br. J. Pharmacol..

[18] Torres-León C., Ventura-Sobrevilla J., Serna-Cock L., Ascacio-Valdés J.A., Contreras-Esquivel J., Aguilar C.N. (2017). Penta-galloylglucose (PGG): A valuable phenolic compounds with functional properties. J. Funct. Foods.

[19] Alañon M.E., Oliver-Simancas R., Gómez-Caravaca A.M., Arráez-Román D., Segura-Carrtero A. (2019). Evolution of bioactive compounds of three mango cultivars (*Mangifera indica* L.) at different maturation stages analyzed by HPLC-DAD-q-TOF-MS. Food Res. Int..

[20] Soong Y.Y., Barlow P.J. (2006). Quantification of gallic acid and ellagic (*Mangifera indica* L.) kernel and their effects onantioxidant activity. Food Chem..

[21] Priyadarsini K.I., Khopde S.M., Kumar S.S., Mohan H. (2002). Free radical studies fo ellagic acid, a natural phenolic antioxidants. J. Agric. Food Chem..

[22] Sroka A., Cisowski W. (2003). Hydrogen peroxide scavenging, antioxidant and antiradical activity of some phenolic acids. Food Chem. Toxicol..

[23] Berardini N., Carle R., Schieber A. (2004). Characterization of gallotannins and benzophenonederivates from mango (*Mangifera indica* L. cv. Tommy Atkins) peles, pulp and kernels by high-performance liquid chromatography/electrospray ionization mass spectrometry. Rapid Commun. Mass Spectrom..

[24] López-Cobo A., Verardo V., Díaz de Cerio E., Segura-Carretero A., Fernández-Gutiérrez A., Gómez-Caravaca A. (2017). Use of HPLC- and GC-QTOF to determine hydrophilic and lipophilic phenols in mango fruit (*Mangifera indica* L.) and its by-products. Food Res. Int..

[25] Du S., Liu H., Lei T., Xie X., Wang H., He X., Tong R., Wang Y. (2018). Mangiferin: An effective therapeutic agent against several disorders (Review). Mol. Med. Rep..

[26] Quadri F., Telang M., Mandhare A. (2019). Therapeutic and socmetic applications of mangiferin: An updated patent review (patents published after 2013). Expert Opin. Ther. Pat..

[27] Sun J., Li L., You X., Li C., Zhang E., Li Z., Chen G., Peng H. (2011). Phenolics and polysaccharides in major tropical fruits: Chemical compositions, analytical methods and bioactivities. Anal. Methods.

[28] Tesfaye T. (2017). Valorisation of mango fruit by-products: Physicochemical characterization and future prospect. Chem. Process. Eng. Res..

[29] Nguyen N.M.P., Le T.T., Vissenaekens H., Gonzles G.B., Van Camp J., Smagghe G., Raes K. (2019). In vitro antioxidant activity and phenolic profiles of tropical fruit by-products. Int. J. Food Sci. Technol..

