# Supplementary material for: Antiplatelet Activity of Natural Bioactive Extracts from Mango (Mangifera Indica L.) and its By-Products
Source: Antioxidants (Basel). 2019 Oct 29;8(11):517. doi: 10.3390/antiox8110517 (PMC6912241; doi:10.3390/antiox8110517)
Supplement: Supplementary file 1 [file antioxidants-08-00517-s001.pdf]

Table S1. Spectral and chromatographic data regarding to the bioactive compounds identified in mango and its by-products by HPLC-DAD-q-TOF-MS.

Proposed Compounds	m/z experimental (M-H)$^-$	m/z calculated (M-H)$^-$	Molecular formula	Error (ppm)	Score (%)	Standard for quantification
Gallic acid	169.015	169.014	$C_7H_6O_5$	-1.36	99.41	Gallic acid
Galloylglucose	331.068	331.067	$C_{13}H_{16}O_{10}$	-7.4	83.25	Gallic acid
Galloyl diglucoside	493.121	493.120	$C_{19}H_{26}O_{15}$	-0.86	97.75	Gallic acid
Methylgallate	183.030	183.030	$C_8H_8O_5$	-4.59	95.09	Gallic acid
Galloylquinic acid	343.068	343.067	$C_{14}H_{16}O_{10}$	-4.51	90.63	Gallic acid
Digallic acid	321.026	321.025	$C_{14}H_{10}O_9$	-1.00	94.15	Gallic acid
Digalloylglucose	483.079	483.078	$C_{20}H_{20}O_{14}$	-6.04	83.94	Gallic acid
Methyl-digallate	355.041	335.041	$C_{15}H_{12}O_9$	-6.02	86.26	Gallic acid
Digalloylquinic acid	495.079	495.078	$C_{21}H_{20}O_{14}$	-2.57	94.29	Gallic acid
Trigalloylglucose	635.091	635.089	$C_{27}H_{24}O_{18}$	-0.76	88.22	Gallic acid
Tetragalloylglucose	787.101	787.100	$C_{34}H_{28}O_{22}$	3.44	88.48	Gallic acid
Pentagalloylglucose	939.112	939.111	$C_{41}H_{32}O_{26}$	-5.68	89.62	Gallic acid
Hexagalloylglucose	1091.124	1091.122	$C_{48}H_{36}O_{30}$	-2.41	94.41	Gallic acid
Vanillic acid glucoside	329.088	329.088	$C_{14}H_{18}O_9$	-1.20	82.97	Vanillic acid
p-Hydroxybenzoic acid glucoside	299.078	299.077	$C_{13}H_{16}O_8$	-2.64	96.20	Gallic acid
Dihydroxybenzoic acid glucoside	315.073	315.072	$C_{13}H_{16}O_9$	-4.55	92.28	Gallic acid
Hydroxybenzoyl galloyl glucoside	451.089	451.088	$C_{20}H_{20}O_{12}$	-2.70	93.37	Gallic acid
Coumaric acid glucoside	325.093	325.093	$C_{15}H_{18}O_8$	-1.63	93.54	Coumaric acid
Coumaroyl galloyl glucoside	477.104	477.104	$C_{22}H_{22}O_{12}$	-3.81	90.91	Gallic acid
Ferulic acid hexoside	355.104	355.104	$C_{16}H_{20}O_9$	-2.60	96.28	Ferulic acid
Sinapic acid hexoside-pentoside	517.230	517.229	$C_{24}H_{38}O_{12}$	-6.40	80.21	Ferulic acid
Dihydro sinapic acid hexoside-pentoside	519.246	519.245	$C_{24}H_{40}O_{12}$	-4.61	88.79	Ferulic acid
Ellagic acid	300.999	300.999	$C_{14}H_6O_8$	-1.20	98.75	Ellagic acid
Catechin	289.073	289.072	$C_{15}H_4O_6$	-3.77	94.78	Catechin
Quercetin glucoside	463.089	463.088	$C_{21}H_{20}O_{12}$	-2.11	97.02	Quercetin
Quercetin galactoside	463.089	463.088	$C_{21}H_{20}O_{12}$	-0.91	97.95	Quercetin
Quercetin xyloside	433.078	433.078	$C_{20}H_{18}O_{11}$	-0.78	95.52	Quercetin
Quercetin arabinopyranoside	433.078	433.78	$C_{20}H_{18}O_{11}$	-0.59	99.76	Quercetin
Rhamnetin hexoside	477.105	477.104	$C_{22}H_{22}O_{12}$	-1.73	97.39	Quercetin
7-O-galloyltricetilflavan	441.083	441.083	$C_{22}H_{18}O_{10}$	-1.29	98.28	Gallic acid
Mangiferin	421.078	421.078	$C_{19}H_{18}O_{11}$	-1.90	98.02	Mangiferin
Maclurin C-glucoside	423.094	423.093	$C_{19}H_{20}O_{11}$	-1.67	98.48	Mangiferin
Maclurin galloyl glucoside	575.105	575.104	$C_{26}H_{24}O_{15}$	-0.81	99.49	Mangiferin
Maclurin digalloyl glucoside	727.116	727.115	$C_{33}H_{28}O_{19}$	-0.58	99.61	Mangiferin
Iriflophenone glucoside	407.099	407.098	$C_{19}H_{20}O_{10}$	-1.21	98.76	Mangiferin
